# Transcatheter closure of Patent ductus arteriosus in a child with IVC interruption through standard femoral access: a case report

**DOI:** 10.1186/s43044-020-00060-6

**Published:** 2020-07-09

**Authors:** Sanjeev H. Naganur, C. R. Pruthvi, Dinakar Bootla, Krishna Prasad, V. Krishna Santosh, Parag Barwad

**Affiliations:** Department of Cardiology, Post Graduate Institute for Medical Education and Research (PGIMER), Chandigarh, India

**Keywords:** Patent ductus arteriosus, IVC interruption, Congenital venous anomalies, Case report

## Abstract

**Background:**

Portsmann and co. performed the first PDA device closure in 1967. The technique and the devices used have evolved since then and are the first choice in managing anatomically feasible patent ductus arteriosus (PDA) for the last 20 years. Though catheter-based closure of PDA is generally a simple procedure, there are instances when the interventionist faces challenges, especially in smaller children, with syndromic features and venous anomalies even when defects are small and pulmonary artery pressures are normal. Although the femoral vein is the relatively risk-free standard access, internal jugular vein, femoral artery, and transhepatic IVC can be used to close the PDA in different anomalies.

The rare venous anomaly of infrahepatic interruption of the IVC with azygous continuation poses technical challenges when percutaneous closure of PDA was attempted through the standard femoral access.

**Case presentation:**

We report a rare case of PDA device closure in a syndromic child with a short neck having interrupted IVC via femoral-azygous venous approach.

**Conclusion:**

Knowledge of the IVC course and its anomalies should be known to the operator before the percutaneous closure of PDA. Although other approaches are available, femoral vein approach can be used in case of interrupted IVC for percutaneous closure of PDA.

## Background

Portsmann and co. performed the first PDA device closure in 1967 [1]. The technique and the devices used have evolved since then and are the first choice in managing anatomically feasible small patent ductus arteriosus (PDA) for the last 20 years [2]. For the past 20 years, percutaneous closure of PDA has become standard practice with a high success rate. The incidence of interrupted IVC is rare which occurs in 2 to 30/1000 population with congenital heart diseases. The knowledge of this anomaly is essential for an interventional cardiologist because it can pose difficulties while attempting percutaneous procedures through a femoral vein.

## Case presentation

A 5-year-old girl was referred for further cardiac evaluation, as she was found to have a systolic murmur on routine cardiac examination by her general pediatrician. Her family did not give any history of recurrent pneumonia or cyanosis, although she was underweight for her age. Her dysmorphism and the mild developmental delay were under evaluation by the concerned experts. She weighed 10 kg and was 90-cm tall. On cardiac examination, she had mild cardiomegaly and a machinery murmur.

There was mild cardiomegaly and increased pulmonary blood flow on chest radiograph with ECG not showing any major abnormalities. An echocardiogram showed hemodynamically significant PDA of size 3.5 mm and normal pulmonary artery pressures. As the child had otherwise normal intracardiac findings and was not very cooperative, we could not appreciate the exact course and drainage of the IVC. Because of these findings and the fact that we did not anticipate any major systemic venous anomalies, the child was electively posted for percutaneous transcatheter closure of PDA.

**Fig. 1:**
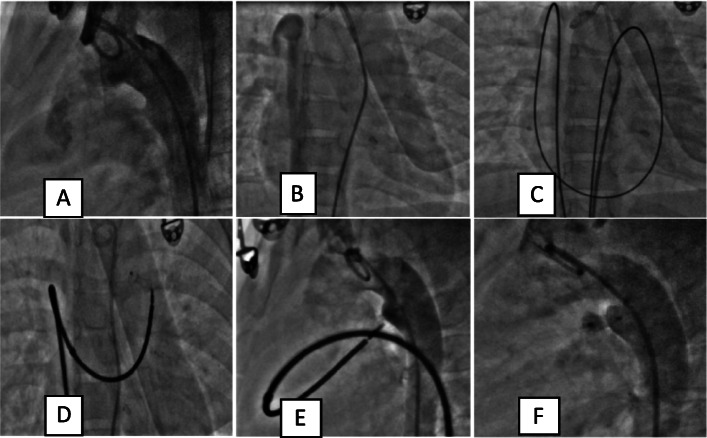
**a** Contrast injection through pigtail in the arch of aorta showing type A PDA. **b** Hand injection of contrast into infrahepatic IVC shows filling of RA via the azygous vein and SVC. **c** Exchange length Amplatzer extra stiff wire used to cross IVC, azygous vein, SVC, RA, RV, MPA, PDA, and descending thoracic aorta (des TA) in sequence. **d** ADO I 6 × 4 mm Cocoon device across PDA. **e** Contrast injection in des TA showing no contrast flow across PDA before release. **f** Contrast injection in descending TA after device release showing no contrast leak into PA suggestive of complete occlusion of PDA

As per our unit’s policy, catheterization and the device closure were performed under intravenous sedation. The standard digital palpation was used to establish the right femoral vein (max 6Fr) and right femoral artery (max 5 Fr). A descending thoracic angiogram (DTA) using 5 Fr pigtail in lateral view showed a 3.5-mm PDA with L → R shunt with good aortic ampulla (Fig.1a in the panel), decided to use 6/4 ADO I device through standard venous approach to close the defect. When we were unable to enter the right atrium (RA) using a 5 Fr multipurpose (MPA) catheter, we suspected IVC interruption. An IVC angiogram showed interrupted infrahepatic IVC with azygous continuation into the superior vena cava (SVC) (Fig. 1b in the panel). The option of internal jugular venous access was considered difficult due to short neck and we did not have any experience of transhepatic access then. We thought to proceed with the standard approach using soft hardware rather than arterial approach to avoid arterial trauma. Arteriovenous loop formation was our back up plan. A 5.0 Fr multipurpose (MPA) (COOK^TM^) catheter was used to enter the pulmonary artery. The pulmonary artery pressures were normal (30/12, mean 18 mmHg). The same catheter was used to cross PDA using straight tip guidewire (TERUMO^TM^) which was exchanged with extra stiff exchange length wire (AMPLATZER^TM^). This was followed by implantation of 6/4 ADO I (COCOON^TM^) through a 6 Fr delivery system manipulated with the utmost care, avoiding injury to the various chambers especially at azygous vein—SVC junction and pulmonary artery to PDA vessels on the way. As there was no residue on repeat DTA angiogram and echo did not show any flow acceleration in the pulmonary artery (LPA) and DTA, device was released. Post device release echo did not show any flow acceleration in the pulmonary artery (LPA) and DTA (Fig. 1c–f in the panel). The procedure was uneventful. Hemostasis was achieved with digital compression and was monitored in the ICU for 12 h with subsequent uneventful stay.

## Discussion

Anomalies of inferior vena cava are uncommon in the absence of lateralization defects. The incidence varies from 0.3 to 2% in patients with normal visceroatrial situs [3] to 90% in those with heterotaxy syndromes [4]. Embryologically, inferior vena cava is formed from five different venous systems. Caudal to cranial, these are the posterior cardinal veins (draining the posterior part of the body), the right supra cardinal veins, the subcardinal veins (draining the mesonephros or urogenital system), the hepatic segment of IVC (formed by small vessels in the dorsal body lateral to the fold of mesentery), and the hepatic veins. The subcardinals, supracardinals, posterior cardinals, and vitelline veins (hepatic veins) initially begin as bilateral structures but remain only on the right side and involute on the left side with the growth of the embryo. Absence of the hepatic segment of IVC with azygous continuation into the right or left SVC leads to interrupted IVC. Very rarely the interrupted IVC may continue to both the right and left SVC as bilateral azygous veins [5].

The azygous vein is formed by the confluence of the suprarenal segment of the right supra cardinal vein and the cephalic part of the right posterior cardinal vein. It starts from the right lumbar or right renal vein, passes through the diaphragm till the fourth thoracic vertebrae where it arches anteriorly to open into the SVC [5]. IVC interruption leads to the enlargement of azygous vein which ultimately drains the abdomen and the lower part of the body into the superior vena cava. Although, isolated interruption of the IVC is usually asymptomatic, the clinical importance lies in planning the surgical procedures (Bidirectional Glenn and modified Fontan operations), during cardiac catheterization (device closure as in the index case), radiofrequency catheter ablations, inferior vena cava filter placement, and temporary pacing through transfemoral route.

Interrupted IVC poses technical challenges [6] during transcatheter closure of PDA and may require a change of access site to the internal jugular vein from the femoral vein [7]. The jugular venous route is associated with the risk of pneumothorax, may need intubation in a small child to avoid airway compression, may limit the sheath size, and can be disastrous in an already heparinized child.

Other options include transhepatic IVC access, but it is more invasive and needs experienced hands. The use of ADO II via the femoral artery is also reported to be convenient [8], but we wanted to avoid trauma to the artery with the use of bigger sheaths. We did not attempt the internal jugular route as the neck was short and anticipated complications in a heparinized child, also it might have needed general anesthesia considering the age and small size of the child. We continued with the femoral venous access as it allows usage of larger delivery sheath if needed. We found standard femoral access with femoral vein-azygous vein route safe and feasible, time convenient for crossing the PDA, and its closure by the device. Although the patient had not yet shown any major complications related to IVC anomaly, she may be at risk of bilateral venous insufficiency and deep venous thrombosis.

## Learning points

1. We should look for vascular anomalies like that of systemic veins when we have a syndromic child with any congenital heart defect.
2. In a child with a short neck, interrupted IVC, we can still use the standard femoral venous approach to negotiate the PDA using soft catheters and guidewires.
3. The arterial approach may be reserved for difficult cases; after femoral vein, internal jugular venous access has been tried, to minimize trauma and bleeding complications.

## Conclusion

To our best of our knowledge, this is the first report in which PDA found in interrupted inferior vena cava is closed via standard femoral venous approach without arterio-venous loop in a child of 10 kg. Interventional cardiologists should be aware of this rare congenital anomaly before cardiac catheterization to consider other than the femoral vein access, although IVC interruption is not a contraindication for femoral access to proceed with the procedure.

## Data Availability

The datasets used and/or analyzed during the current study are available from the corresponding author on reasonable request.

## Abbreviations

ADO: Amplatzer duct occlude
DTA: Descending thoracic angiogram
IVC: Inferior vena cava
MP: Multipurpose
PDA: Patent ductus arteriosus
RA: Right atrium
SVC: Superior vena cava
Fr: French
